# Supplementary material for: Higher serum dehydroepiandrosterone sulfate protects against the onset of depression in the elderly: Findings from the English Longitudinal Study of Aging (ELSA)
Source: Psychoneuroendocrinology. 2016 Feb;64:40–6. doi: 10.1016/j.psyneuen.2015.11.005 (PMC4712651; doi:10.1016/j.psyneuen.2015.11.005)
Supplement: Supplementary file 1 [file mmc1.docx]

**Higher serum dehydroepiandrosterone sulfate protects against the onset of depression in the elderly: findings from the English Longitudinal Study of Ageing (ELSA)**

**Luis H Souza-Teodoro^1,3^; Cesar de Oliveira^1^; Kate Walters ^2^; Livia A Carvalho^1^**

^1^Department of Epidemiology and Public Health, University College London, London, UK;

^2^Research Department of Primary Care and Population Health, UCL and Medical Research Council General Practice Research Framework, London, UK

^3^Chronopharmacology Laboratory, Institute of Biosciences, University of São Paulo, São Paulo, Brazil

**Running title:** DHEA(S) protects against the onset of depression

***Corresponding author:** Livia A Carvalho, Department of Epidemiology and Public Health, University College London, 1-19 Torrington Place, London WC1E 6BT, UK.

Tel: (44) 207 679 5973; Fax: (44) 207 916 8542; email: [livia.a.carvalho@ucl.ac.uk](mailto:a.ronaldson@ucl.ac.uk)

Abstract = 201 words

Main Text = 4284 words

Number of Figures and Tables = 4

Supplementary Tables = 2

**Abstract**

Depression is one of the major causes of disability worldwide, but the complete aetiology of depression is not fully understood. Dehydroepiandrosterone (DHEA) and its sulphated form DHEA(S) have been associated with mood and healthy ageing. Associations with mental illness over the middle to late years of life have not yet been extensively investigated in large, western community-dwelling samples. The aim of this study was to investigate whether low DHEA(S) levels are associated with the development of depressive symptoms in a large longitudinal cohort study of older men and women. We assessed data from English Longitudinal Study of Ageing (ELSA) to evaluate the association of DHEA(S) levels and depressive symptoms measured by Center for Epidemiologic Studies Scale (CES-D) at baseline (n = 3083) and at 4-year follow-up (n = 3009). At baseline, there was an inverse association between DHEA(S) and depressive symptoms (B = -0.252, p = 0.014). Adjustments for physical illnesses, impairments in cognitive function and health behaviours abolished this association (p = 0.109) at baseline. Decreased DHEA(S) levels at baseline also predicted incident depression at 4-year follow-up (B= -0.332, p <0.001). In conclusion, higher DHEA(S) levels were associated with reduced risk of developing depressive symptoms in both men and women.

**Key Words:** adrenal gland, antiglucocorticoid, mental health, psychoneuroimmunology, ageing, psychological stress, biomarkers

**1.0 Introduction**

Depression is the second major cause of disability worldwide affecting around 350 million people (WHO, 2009). Although the precise aetiology of depression is complex and remains unclear, there is evidence that the prevalence of depressive disorder increases with age (Samaras et al., 2013) and affects around 17% of the elderly population (Luppa et al., 2010).

Dehydroepiandrosterone (DHEA) is a steroid hormone mainly synthesized in the adrenal glands in response to adrenocorticotrophic hormone, although *de novo* synthesis in the brain has also been described (Maninger et al., 2009; Sugaya et al., 2015). DHEA and its sulphated form DHEA(S) are the more prevalent circulating steroid hormones, acting as precursors of the sexual hormones, as a neurosteroid, and it is hypothesised to present opposite effects to glucocorticoids (Maurice et al., 1996; Kimonides et al., 1999; Binello and Gordon, 2003; Maninger et al., 2009; Moriguchi et al., 2013). DHEA has been described as a modulator of neurotransmission as it can affect serotonin, γ-amino butyric acid (GABA), glutamate, and dopamine levels (Kimonides et al., 1998; Robichaud and Debonnel, 2004; Traish et al., 2011). Studies demonstrate that DHEA presents a strong age-related decline (Berr et al, 1996; Kroboth et al., 1999), and thus may help explain age related increases in depression.

The role of DHEA(S) in depression is still poorly understood. In clinically diagnosed depressed patients the role DHEA(S) plays is still inconclusive, as studies showing increased (Assies et al., 2004; Morita et al., 2014), decreased (Kurita et al., 2012; Lopes et al., 2012) and no changed levels have been found (Erdinçler et al., 2004). In the population, most cross-sectional studies report that lower DHEA(S) levels are associated with poor mental health (Haren et al., 2007; Wong et al., 2011; Michikawa et al., 2013) although no association has also been reported (ó Hartaigh et al., 2012). The role of gender on this association is still inconclusive (Berr et al., 1996; Michikawa et al., 2013).

Few prospective studies have been conducted and are comparatively small in sample size or have analysed women or men exclusively. These studies seem to point towards an association of lower DHEA(S) levels with incident depression (Berr et al, 1996; Goldman and Glei, 2007; Michikawa et al., 2013; Veronese et al., 2015). The aim of this study was to investigate whether lower DHEA(S) levels predicts the development of depressive symptoms in a large community-dwelling sample of older men and women.

**2.0 Methods**

**2.1 Study Population**

The English Longitudinal Study of Ageing (ELSA) is a longitudinal study of men and women aged 50 and over, and is representative of people living in England (Steptoe et al., 2013). Objective and subjective data relating to health and disability, biological markers of disease, economic circumstances, social participation, networks and well-being were collected. ELSA aims to measure outcomes across a wide range of domains and to provide high-quality multidisciplinary data that can shed light on the causes and consequences of outcomes of interest. Wave 1 was collected in 2002/3, and participants are followed-up every 2 years, with a nurse visit and biomarker assessment every four years (http://www.ifs.org.uk/ELSA). Participants gave full informed consent to participate in the study and ethical approval was obtained from the London Multi-centre Research Ethics Committee. For the purposes of the present analyses, data collected at wave 4 (2008/9) were used as the baseline.

For the present study, 10,601 people participated in the study. Since 2008, 1788 people had died by 2013 follow-up period and were therefore not included in the analysis. 2309 were excluded due to missing depressive symptoms scores at baseline. Moreover, 3,421 participants were excluded because there were no information about DHEA(S) and/or covariates. We have excluded participants with doctor-diagnosed dementia. Thus, at baseline there were 3083 people. Seventy-four participants did not have CESD scores at follow-up. At follow-up the analytical sample consisted of 3,009 participants eligible for analysis. Selection of the analytical sample is represented in Figure 1.

Compared to those study members who featured in the analytical sample, people with missing data were more likely to be female (X^2^ = 20.75, P < 0.001), smokers (X^2^ = 26.27, P < 0.001), on the lowest tertile of wealth (X^2^ = 12.20, P < 0.001), to report higher depressive symptoms (total sample 1.40 ± 1.90, analytical 1.11 ± 1.70, p < 0.001), be inactive (X^2^ = 41.71, P < 0.001), and have higher frequency of cognitive impairment (total score total sample 29.77 ± 6.99, analytical sample 31.21 ± 5.73, p < 0.001). However, no more likely to have diabetes, cardiovascular disease, stroke or cancer (X^2^ = 0.199, P = 0.348), be obese (X^2^ = 0.83,
P = 0.402) or consume alcohol (X^2^ = 4.337, P = 0.114).

-----------------------------------------------Figure 1-----------------------------------------------

**2.2 Assessment of Depressive Symptoms**

Depressive symptomatology was measured using the eight-item Center for Epidemiological Studies-Depression (CES-D) scale, a widely used measure that identifies people “at risk” of depression in population surveys (Radloff, 1977; Turvey et al, 1999). The psychometric values of the eight-item CES-D are comparable to those of the full 20-item CES-D (Steffick, 2000). We derived a summary CES-D score by adding responses to all eight dichotomous questions (possible range: 0 - 8). To exclude cases of elevated depressive symptoms that are possible cases of clinical depression at baseline, we dichotomized the summary score around the cut point of four or higher, a conservative threshold that corresponds to the cut point of 16 or higher on the 20-item CES-D (Steffick, 2000) in our longitudinal analysis.

**2.3 Assessment of DHEA(S)**

Blood samples were taken from willing ELSA core members, except those who had a clotting or bleeding disorder (e.g. haemophilia or low platelets), had ever had a fit, were not willing to give their consent in writing or were currently on anticoagulant drugs (e.g. warfarin therapy).

DHEA(S) was only collected at baseline. The analysis of the blood data was carried out in the Royal Victoria Infirmary (Newcastle-upon-Tyne, UK). The analysis of DHEA(S) levels from serum was performed using the Roche DHEA(S) assay that is a competitive immunoassay using electrochemiluminescence technology (analytical range: 0.003 - 27 µmol/L). During the first incubation step DHEA(S) binds with a biotinylated monoclonal DHEA(S)-specific antibody. During the second incubation a DHEA(S) derivative labeled with a ruthenium complex occupies the remaining free binding sites on the biotinylated antibody. The entire complex becomes bound to the streptavidin-coated microparticulate solid phase via interaction of biotin and streptavidin. The reaction mixture is aspirated in to the measuring cell where the microparticles are magnetically captured onto the surface of the electrode. Unbound substances are removed using Procell. Application of a voltage to the electrode induces chemiluminescent emission that is measured by a photomultiplier. Results are determined by measuring the electrochemiluminescence signal obtained from the reaction product of the sample against a calibration curve generated by 2-point calibration and a master curve provided via the reagent barcode. Detailed information on the technicalities of the blood analysis, the internal quality control and the external quality assessment for the laboratory have been described elsewhere (Graig et al., 2006).

**2.4 Measurement of covariates**

Participants’ age, gender and BMI were assessed during a face-to-face visit in the home. Height and weight, which were assessed by a nurse, were used to calculate body mass index (BMI, kg/m^2^). Socioeconomic status was indexed by total household wealth, including financial wealth (savings and investments), the value of any home and other property (less mortgage), the value of any business assets and physical wealth such as artwork and jewelry, net of debt. Wealth is the most robust indicator of socioeconomic circumstances in ELSA (Banks et al., 2003). Cohabitation was defined as currently living alone or not. Cognitive function was assessed using a test of memory and executive function (Steel et al., 2003). Self-reported health behaviours included smoking status (current, ex-smoker/never), frequency of alcohol consumption in the past year (rarely (< 1 week), occasionally (> 1/week), frequently (> 1/day), and physical activity during leisure time, recorded as participation in vigorous, moderate, mild or sedentary activities (more than once per week, once per week, 1 - 3 times per month, hardly ever) (Hamer et al., 2009). Chronic illness was assessed as self-reported doctor diagnosed diabetes mellitus (self-report or medication), cancer, stroke, and cardiovascular diseases (“Yes”/”No”).

**2.5 Statistical analysis**

All analyses were conducted using SPSS, version 22 (SPSS Inc., Chicago IL). We normalized the distribution of DHEA(S) by natural logarithmic transformation and used linear regression to estimate standardized multinomial linear regression coefficient (B) and 95% confidence interval (CI) for depressive symptoms at baseline (2008/9). We treated DHEA(S) as continuous variable. To assess the association between the log of the mean DHEA(S) and depressive symptoms at 4-year follow-up, we performed multinomial linear regression analysis of the CES-D scores at 4-year follow-up (2012/13) with adjustment for depressive symptoms at baseline (2008/9). To further explore persistent depressive symptoms over the 4 years (2008/9-2012/2013), multinomial logistic analysis were performed using the CES-D scores (> 4) at baseline and at 4-year follow-up. We estimated unadjusted models that were adjusted for age, sex, marital status, antidepressant use, wealth, cognitive impairment, smoking status, BMI, alcohol consumption, physical activity and history of chronic illnesses (cardiovascular disease, diabetes, stroke and cancer).

**3.0 Results**

**3.1 Demographic characteristics**

Table 1 represents information on socio-demographic and clinical characteristics of the 3083 participants at baseline. Fifty four percent of the study sample were women. The majority of participants were at the richest tertile, most people did not smoke, consume alcohol occasionally, conducted vigorous physical activity once a week. Eleven percent of people were depressed at baseline, and 10% at 4-years follow-up.

--------------------------------Table 1----------------------------------

**3.2 DHEA(S) and depressive symptoms are negatively associated at baseline**

To determine whether there was a cross-sectional relationship between serum levels of DHEA(S) and current depressive symptoms, we performed linear regression analysis between DHEA(S) (log-transformed continuous values) with CES-D scores at baseline. DHEA(S) was 17% lower in people with high depressive symptoms compared to those with low depressive symptoms. In our basic model, we corrected for age and gender at baseline. Subsequently, corrections for socio-demographic, health characteristics and health behaviours were performed. There was an association between DHEA(S) and depressive symptoms at baseline after adjustment for socio-demographic factors. However, the association was attenuated and became non-significant (p = 0.109) after corrections for all potential confounders were considered (Table 2).

There was no interaction between DHEA(S) and gender in our analysis. However, as previous studies have found associations of DHEA(S) and depressive symptoms to be gender-specific we have conducted these associations separately in men and women and added this information in supplementary tables. In our study, women had significantly lower levels of DHEA(S) (men = 3.09 ± 2.06 versus women = 1.84 ± 1.29, p < 0.001). At baseline, despite the association in men (but not in women) in our basic model (p = 0.015), there was no association between DHEA(S) neither in men (p = 0.187, supplementary table 1A) nor in women (p = 0.239, supplementary table 1B) when all potential confounders were considered.

--------------------------------Table 2----------------------------------

**3.3 Low DHEA(S) levels predict depressive symptoms at 4-year follow-up**

To investigate whether DHEA(S) levels are associated with future depressive symptoms at 4-year follow-up, linear regressions were performed with CES-D scores at 4-year follow-up. One hundred and sixty four new cases of depression were detected. Those who developed depression had 19% lower DHEA(S) levels compared to those who did not develop depression. In our basic model, we corrected for age, gender and depressive symptoms at baseline. Subsequently, corrections included socio-demographic, health characteristics (B = -0.331, CI -0.518, -0.145, p = 0.001) and health behaviours. Adjustment for health characteristics or health behaviours (B = -0.332, CI -0.520, -0.145, p = 0.001) did not modify the association between DHEA(S) and depressive symptoms (Table 3). When stratified by gender, DHEA(S) was associated with future development of depressive symptoms at 4-year follow-up in men (p = 0.004, supplementary tables 2A) and in women (p = 0.025, supplementary table 2B).

--------------------------------Table 3----------------------------------

**3.4 Exploratory analysis**

DHEA(S) has been previously observed to decline in those who have a persistent major depressive episode (Mocking et al., 2015). We have ran a nominal regression to investigate whether people with lower levels of DHEA(S) were at higher risk of having higher depressive symptoms at both waves, compared to having depression at only one wave or none. Our data showed that people who had persistent depression did not have lower levels of DHEA(S), when compared to people who did not have persistent depression (-0.078%, B = -0.393, CI 0.354, -1.287, p = 0.233).

--------------------------------Table 4----------------------------------

We also explored whether people who took antidepressants had different DHEA(S) levels, than people who did not take antidepressants in the past two years. Levels of DHEA(S) did not differ between those who had or not taken antidepressants in the past two years (Yes, n = 98, 2.25 ± 1.64 versus No, n = 2985,
2.41 ± 1.8 µmol/L, p = 0.445 respectively). Between those who were on antidepressants, DHEA(S) levels also did not differ whether people had low or high depressive symptoms (low n = 60, 2.32 ± 0.25, high n = 38, 2.33 ± 0.22, p = 0.52).

4.0 Discussion

In this large longitudinal cohort study we demonstrated that low levels of DHEA(S) predicts future depressive symptoms in both men and women. In our analyses, the longitudinal associations observed between DHEA(S) and depressive symptoms were independent of age, gender, economic circumstances and health behaviours and characteristics.

4.1 DHEA(S) levels and depressive symptoms

Our study is the largest to date to investigate the relationship between DHEA(S) and future depressive symptoms in a community-dwelling sample of both men and women. The longitudinal findings of our study are supported by other smaller prospective studies that showed lower DHEA(S) levels associated to poor mental health. A recent paper reported higher DHEA(S) levels to be protective for the onset of depression irrespective of gender (Veronese et al., 2015). In their study, DHEA(S) was also inversely associated with incident severe depression but this association was exclusively observed in men (Veronese et al., 2015). In our study severity of depressive symptoms could not be assessed as we only used a brief depressive symptoms questionnaire and therefore results cannot be directly comparable. Mazat et al. (2001) found an association between DHEA(S) levels and mortality in men who smoke, and an association with depressive symptomatology in women. Other studies that have investigated DHEA(S) and incident depressive symptoms found it to be gender specific (in men, Michikawa et al., 2013) (in women Berr et a., 1996; Yaffe et al., 1998; Mazat et al., 2001). In addition, there are reports of no longitudinal association in either gender (ó Hartaigh et a., 2012). Herein, we did not find DHEA(S) and gender interaction in the association with depression. Explanations for differences in the association related to gender are debatable and not yet clear.

DHEA(S) was inversely associated with current depressive symptoms cross-sectionally in the basic model, but adjustment for chronic illness and health behaviours abolished the association. When compared to other population studies, our study agrees with others who showed no association of DHEA(S) and depression at baseline (Erdinçler et al., 2004, T’Sjoen et al., 2005; Hsu et al., 2009; ó Hartaigh et al., 2012). The role of DHEA(S) in depression is still unclear as inverse associations have also been reported in the population (Wong et al., 2011, Michikawa et al., 2013). In clinically diagnosed depressed patients, a large cross-sectional study also did not find an association (Phillips et al., 2011). In contrast, a recent cross-sectional meta-analysis confirmed findings of a lower DHEA(S) in people with clinical major depression, however, adjustment for ethnicity abolished the association in Caucasians (Hu et al., 2015). The explanation why we only found longitudinal associations is not clear. It is possible that persistently low DHEA(S) is influencing the ageing process so those with low DHEA(S) at baseline experience greater declines over the follow-up e.g. in frailty over and above their chronic diseases and lifestyle, leading to greater depressive symptoms. Indeed, Mazat et al. (2001) has measured DHEA(S) overtime in the population and shown a bigger impact on health associated with greater DHEA(S) decline. Moreover, as DHEA(S) declines with age (Kroboth et al., 1999), it is possible that that lower levels of DHEA(S) become more predominant and detrimental to physical and mental health as one ages (Berr et al., 1996; Bauer et al., 2005; Buford and Willoughby et al., 2008). Low DHEA(S) has also been associated with poor physical illness (Berr et al., 1996). It is thus possible that people who had lower levels of DHEA at baseline developed more physical illnesses, which then led them to experience more depressive symptoms at follow up.

In our exploratory analysis, we did not find an association between DHEA(S) and persistent depression. This association was contrary to a recent study which demonstrated how DHEA(S) remains equally altered between episodes and may predict future recurrence (Mocking et al., 2015). Our study differs from that of Mocking et al. (2015), who have used a clinical patient group of people with a major depression diagnosis, whilst we used a population cohort. The difference in population may explain the confounding results. It is also possible that we did not find an association with persistent depression due to the low number of people in our group (only 178 people). Among clinically depressed patients, DHEA(S) role remains unclear as no changed (Erdinçler et al., 2004), increased (Assies et a., 2004; Morita et a., 2014) or decreased (Lopes et al., 2012) levels have been reported.

4.2 Sex differences

An interaction between gender and DHEA(S) was not found here, despite the fact that women had significantly lower levels of DHEA(S). Population cohorts show controversial data regarding the influence of gender in the association between DHEA(S) and depression. Low DHEA(S) levels are associated to poor health in men (Tilvis et al., 1999; Goldman and Glei, 2007, Wong et al., 2011; Michikawa et al., 2013), while others find an association in women (Yaffe et al., 1998; Morrison et al., 2000, Haren et al., 2007), and others in neither (Glei et al., 2004; Hsu et al., 2009, ó Phillips et al., 2011, ó Hartaigh et al., 2012, Hu et al., 2015). Mazat et al. (2011) found low DHEA(S) levels to be associated with mortality in men, but depressive symptomatology was only associated with women. Michikawa et al. (2013) found an association between depressive symptoms in men, but not in women, while Berr et al. (1996) found the inverse. Several studies only investigated associations exclusively in one gender (men only, T’Sjoen et al., 2005; Wong et al., 2011) (women only, Yaffe et al., 1998; Barrett-Connor et al., 1999; Haren et al., 2007).

One hypothesis to why our results differ from others may be ethnical composition of our sample. A recent meta-analysis despite concluding that DHEA(S) is associated with clinical major depression (Hu et al., 2015), did not find similar associations in Caucasians or Asians in ethnicity-stratified analysis. Morrison et al. (2001) has described racial differences among the relationship between DHEA(S) levels and depressive symptoms. In our sample, non-white women had significantly lower levels of DHEA(S) as compared to white women (non-white 1.39 ± 0.15, white 1.85 ± 0.029, p = 0.05, n = 32); whereas there was no difference between white and non-white men . We could not find an association of depressive symptoms and DHEA(S) in non-white people however lack of power is an issue. Morsink et al. (2007) reported a difference in the levels of testosterone between white and black women. DHEA is biotransformed to testosterone, and this hormone improves depressive symptoms (Zarrouf et al., 2009). DHEA has also shown to induce pain acutely, but to have antinociceptive action in the long-term, an effect partly related to analgesic effects of testosterone (Kibaly et al., 2008). Unfortunately, sex differences in analgesic effects might be relevant, but could not be investigated here. Diet may also be another explanation. DHEA(S) is a precursor of estrogen and androgens. Countries that consume more soy-based products may contribute to extra sources of estrogen in women. DHEA(S) and soy diet is inversely associated with depressive symptoms, perhaps due to the role of increased estrogen levels on mental wellbeing (Nagata et al., 2000). Adjustments for chronic illnesses may also be relevant to explain differences in findings. In stratified analysis by chronic illnesses, DHEA(S) was associated to depression cross-sectionally in our sample only in women who were chronically ill (full-adjusted B = 3.021, p = 0.003). Other reasons for the differences are not clear. Direct comparisons between the levels of DHEA(S) are difficult as differences in social, demographic, health behaviours and characteristics, culture and environment could influence DHEA(S) levels.

4.3 Contribution of health characteristics and behaviour

In our analysis, health characteristics and behaviours reduced the association, which in our baseline cross-sectional analysis became non-significant. One explanation might be that common mental health disorder is a consequence of lower DHEA(S) resulting from chronic pain, and disability associated with physical illness. In fact, there is evidence showing that chronic stress diminishes DHEA(S) levels (Lennartsson et al, 2015). In this sense, chronic illnesses might affect hippocampal brain cells and lymphocytes to become more vulnerable to the cytotoxic and modulatory effects of glucocorticoids with age (Kimonides et al., 1999). Moreover, chronic illnesses can lead to a degenerative change that may be explained by a decline in DHEA (McEwen et al., 1997; Bauer al., 2008; Bauer et al., 2009). Another explanation might be that DHEA(S) acts as a risk factor to influence both psychological and physical health, in a common cause hypothesis. In this study, we could not dissect these mechanisms as DHEA(S) was only collected at baseline. The reason as to why chronic illnesses decrease the association between DHEA(S) and depression is not clear. Conflicting information is observed in the literature. DHEA(S) has been associated with physical illness, and with frailty in the elderly, but a positive association between DHEA(S) and cognitive impairment was observed in women only (Morrison et al., 2000).

In our study, health behaviours further reduced the cross-sectional association between depressive symptoms and DHEA(S). Disputed information is observed in the literature regarding the influence of these covariates in the DHEA(S) and depression relationship. Smoking did not modify DHEA(S) levels in the study of Chehab et al. (2007), whilst other studies report smoking decreases DHEA(S) levels (Orentreich et al., 1992; Hsiesh et al., 1998). Obesity has shown to present an inverse association to DHEA(S) levels (Tchernof and Labrie, 2004). Although, one study in major depressed patients reported that neither smoking nor BMI affect DHEA(S) and depression association (Markopoulou et al., 2009). Some studies have demonstrated that alcohol consumption increases DHEA(S) levels (Dorgan et al., 2001; Sierksma et al., 2004); this effect might be dependent on the type of alcoholic drink, as Hirko et al. (2014) observed a positive correlation with beer intake, but not with other alcoholic drinks. Physical exercise was observed to increase DHEA levels, but DHEA(S) was increased only in women. One hour after the exercise, hormone levels returned to values prior to exercise (Heaney et al., 2013). Different results between DHEA and DHEA(S) might occur once stimulus activating sympathetic nervous system could affect DHEA sulfatase or sulfotransferase, thus altering DHEA(S) levels (Kizildere et al., 2003). Besides, body fat distribution is also a possible bias of exercise that could explain controversial data (Straub et al., 2008).

4.4 Mechanisms of association

The role of DHEA and/or DHEA(S) in relation to depression might partially rely upon a relationship with cortisol (Mocking et al., 2015; Sugaya et al., 2015). DHEA is produced in the adrenal cortex and has a variety of physiological functions, including anti-glucocorticoid effects (Maninger et al., 2009). There is preliminary evidence that DHEA lower cortisol levels in the periphery, providing further support for the antiglucocorticoid effect of DHEA. Besides, DHEA acts as an antagonist to cortisol by interacting with the glucocorticoid receptor (Kimonides et al., 1999), and DHEA promotes neurogenesis by antagonizing corticosterone neurotoxicity (Karishma and Herbert, 2002). Since persistently high levels of cortisol levels are associated with increased risk of depression, DHEA may have neuroprotective effects in the brain diminishing the liability for cortisol to damage neurons and decreasing the risk for psychopathology.

Moreover, DHEA may also impacts depression directly. Administration of DHEA increases both DHEA and its sulphated form without affecting cortisol levels (Wolkowitz et al., 1997), what suggests specific roles attributed to both DHEA and DHEA(S) themselves. Studies with animal models show that treatment with DHEA (Moriguchi et al, 2013) and/or DHEA(S) (Malkesman et al., 2009) ameliorate depressive behaviour via a direct action of DHEA stimulation in sigma receptors (Maurice et al., 1996), which in turn modulate noradrenergic and serotonergic neurotransmission. In fact, DHEA(S) concentration in the brain is higher than its plasmatic levels even after adrenalectomy and gonadectomy, emphasizing the relevant roles of DHEA(S) as a neuromodulator (Maninger et al., 2009).

4.5 Limitations

Our study has both strengths and limitations. We used a large representative community-dwelling sample of older men and women, an internationally validated questionnaire specific for depressive symptoms, a population where supplementation of DHEA(S) is uncommon, and a prospective design. On the other hand, we only assessed DHEA(S) once at baseline and thus cannot further understand mediation/moderation effect of health behaviours. Lack of knowledge of current antidepressant use could create a bias, although this seems unlikely to have had a substantial effect. Only 3% of our population reported having used antidepressants in the last two years; levels of DHEA(S) also did not differ between those who had or not taken it in the past. Although we have adjusted for depression at baseline on our prospective analysis, we cannot exclude that reverse causation (impact of disease on DHEA(S)) does not play a role as individuals with depressive symptoms prior to the date of collection could have had lower levels of DHEA(S). Finally, it is possible that in our study what we show is a conservative account of the real association due to selection bias. People who gave blood were more likely to be healthier than those who did not.

4.6 Conclusions

Our findings provide evidence that lower DHEA(S) levels predict incident depressive symptoms in both men and women. Future research is needed to further examine whether DHEA(S) levels may be used to identify those who would particularly benefit from targeted prophylactic treatment, and to further understand the role of gender.

**Acknowledgements**

Livia A. Carvalho is funded by the British Heart Foundation grant number RG/10/05/28296 and by the MRC Immunopsychiatry Consortium grant number 520373.

**Conflict of Interest**

The authors have no conflict of interest to disclose.

**References**

Assies, J., Visserb, I., Nicolsonc, N. A., Eggelted, T. A., Wekkinga, E. M., Huysera, J., Lieversee, R., Schenea, A. H. (2004). Elevated salivary dehydroepiandrosterone-sulfate but normal cortisol levels in medicated depressed patients: preliminary findings. Psychiatry Research 128(2): 117-122.

Banks, J., Karlsen, S., Oldfield, Z. (2003). Socioeconomic position. In Health, Wealth and lifestyles of the older population in England: The 2002 English Longitudinal Study of Ageing, ed. J. Banks, R. Blundell, C. Lessof, M. Marmot and J. Nazroo. London: Institute for Fiscal Studies.

Barrett-Connor, E., von Mühlen, D., Laughlin, G. A., Kripke, A. (1999). Endogenous levels of dehydroepiandrosterone sulfate, but not other sex hormones, are associated with depressed mood in older women: the Rancho Bernardo Study. Journal of the American Geriatrics Society 47(6): 685-691.

Bauer, M. E. (2005). Stress, glucocorticoids and ageing of the immune system. Stress 8(1): 69-83.

Bauer, M. E. (2008). Chronic stress and immunosenescence. Neuroimmunomodulation 15: 241-250.

Bauer, M. E., Jeckel, C. M., Luz, C. (2009). The role of stress factors during aging of the immune system. Annals of the New York Academy of Sciences 1153: 139-152.

Berr, C., Lafont, S., Debuire, B., Dartigues, J. F., Baulieu, E. E. (1996). Relationships of dehydroepiandrosterone sulfate in the elderly with functional, psychological, and mental status, and short-term mortality: A French community-based study. Proceedings of the National Academy of Sciences of the United States of America 93(23): 13410-13415.

Binello, E., Gordon, C.M. (2003). Clinical uses and misuses of dehudroepiandrosterone. Current Opinion in Pharmacology 3(6): 635-641.

Buford, T. W., Willoughby, D. S. (2008). Impact of DHEA(S) and cortisol on immune function in aging: a brief review. Applied Physiology Nutrition and Metabolism 33(3): 429-433.

Chehab, O., Ouertani, M., Chaieb, K. Haouala, F., Mahdouani, K. (2007). Hormonal status of cortisol and dehydroepiandrosterone sulphate in an elderly Tunisian population. Comptes Rendus Biologies 330(10): 755-763.

Dorgan, J. F., Baer, D. J., Albert, P. S., Judd, J. T., Brown, E. D., Corle, D. K., Campbell, W. S., Hartman, T. J., Tejpar, A. A., Clevidence, B. A., Giffen, C. A., Chandler, D. W., Stanczyk, F. Z., Taylor, P. R. (2001). Serum hormones and the alcohol-breast cancer association in postmenopausal women. Journal of the National Cancer Institute 93(9): 710-715.

Erdinçler, D., Bugay, G., Ertan, T., Eker, E. (2004). Depression and sex hormones in elderly women. Archives of Gerontology and Geriatrics 39(3): 239-244.

Glei, D. A., Goldman, N., Weinstein, M., Liu, I. W. (2004). Dehydroepiandrosterone sulfate (DHEAS) and health: does the relationship differ by sex? Experimental Gerontology 39(3): 321-331.

Goldman, N., Glei, D. A. (2007). Sex differences in the relationship between DHEAS and health. Experimental Gerontology 42(10): 979-987.

Graig, R., Deverill, C., Pickering, K. (2006). Quality control of blood, saliva and urine analytes. In: Spronston K, Mindell J (eds) Health survey for England 2004, Methodology and documentation, vol 2. The Information Centre: London: 34-41.

Hamer, M., Molloy, G. J., de Oliveira, C., Demakakos, P. (2009). Leisure time physical activity, risk of depressive symptoms, and inflammatory mediators: the English Longitudinal Study of Ageing. Psychoneuroendocrinology 34(7): 1050-1055.

Haren, M. T., Malmstrom, T. K., Banks, W. A., Patrick, P., Miller, D. K., Morley, J. E. (2007). Lower serum DHEAS levels are associated with a higher degree of physical disability and depressive symptoms in middle-aged to older African American women. Maturitas 57(4): 347-360.

Heaney, J. L., Carroll, D., Phillips, A. C. (2013). DHEA, DHEA-S and cortisol responses to acute exercise in older adults in relation to exercise training status and sex. Age 35(2): 395-405.

Hirko, K. A., Spiegelman, D., Willett, W. C., Hankinson, S. E., Eliassen, A. H. (2014). Alcohol consumption in relation to plasma sex hormones, prolactin, and sex hormone-binding globulin in premenopausal women. Cancer Epidemiology, Biomarkers & Prevention 23(12): 2943-2953.

Hsu, H. J., Chen, C. K., Hsiao, C. C., Wu, I. W., Sun, C. Y., Chou, C. C., Lee, C. C., Tsai, C. J., Wu, M. S. (2009). Lack of association between plasma dehydroepiandrosterone sulfate (DHEA-S) levels and depression in hemodialysis patients: a cross-sectional study. Experimental Gerontology 44(11): 733-739.

Hu, Q., Zhang, S.Y., Liu, F., Zhang, Y. L., Zhu, D.M., Zang, Y. Y. (2015). Clinical significance of decreased protein expression of dehydroepiandrosterone sulfate in the development of depression: A meta-analysis. Journal Of Affective Disorders 174: 416-423.

Karishma, K. K., Herbert, J. (2002). Dehydroepiandrosterone (DHEA) stimulates neurogenesis in the hippocampus of the rat, promotes survival of newly formed neurons and prevents corticosterone-induced suppression. The European Journal of Neuroscience 16(3): 445-453.

Kibaly, C., Meyer, L., Patte-Mensah, C., Mensah-Nyagan, A. G. (2008). Biochemical and functional evidence for the control of pain mechanisms by dehydroepiandrosterone endogenously synthesized in the spinal cord. The FASEB Journal 22(1): 93-104.

Kimonides, V. G., Khatibi, N. H., Svendsen, C. N., Sofroniew, M. V., Herbert, J. (1998). Dehydroepiandrosterone (DHEA) and DHEA(S)ulfate (DHEAS) protect hippocampal neurons against excitatory amino acid-induced neurotoxicity. Proceedings of the National Academy of Sciences of the United States of America 95(4): 1852-1857.

Kimonides, V. G., Spillantini, M. G., Sofroniew, M. V., Fawcett, J. W., Herbert, J. (1999). Dehydroepiandrosterone antagonizes the neurotoxic effects of corticosterone and translocation of stress-activated protein kinase 3 in hippocampal primary cultures. Neuroscience 89(2): 429-436.

Kizildere, S., Glück, T., Zietz, B., Schölmerich, J., Straub, R. H. (2003). During a corticotropin-releasing hormone test in healthy subjects, administration of a beta-adrenergic antagonist induced secretion of cortisol and dehydroepiandrosterone sulfate and inhibited secretion of ACTH. European Journal of Endocrinology 148(1): 45-53.

Kroboth, P. D., Salek, F. S., Pittenger, A. L., Fabian, T. J., Frye, R. F. (1999). DHEA and DHEA(S): a review. Journal of Clinical Pharmacology 39(4): 327-348.

Kurita, H., Maeshima, H., Kida, S., Matsuzaka, H., Shimano, T., Nakano, Y., Baba, H., Suzuki, T., Arai, H. (2012). Serum dehydroepiandrosterone (DHEA) and DHEA(S)ulfate (S) levels in medicated patients with major depressive disorder compared with controls. Journal of Affective Disorders 146(2): 205-212.

Lennartsson, A. K., Sjörs, A., Jonsdottir, I. H. (2015). Indication of attenuated
DHEA-S response during acute psychosocial stress in patients with clinical burnout. Journal of Psychosomatic Research 79: 107-111.

Lopes, R. P., Grassi-Oliveira, R., de Almeida, L. R., Stein, L. M., Luz, C., Teixeira, A. L., Bauer, M. E. (2012). Neuroimmunoendocrine interactions in patients with recurrent major depression, increased early life stress and long-standing posttraumatic stress disorder symptoms. Neuroimmunomodulation 19(1): 33-42.

Luppa, M., Sikorski, C., Luck, T., Ehreke, L., Konnopka, A., Wiese, B., Weyerer, S., König, H. H., Riedel-Heller, S. G. (2010). Age- and gender-specific prevalence of depression in latest-life – Systematic review and meta-analysis. Journal of Affective Disorders 136(3): 212-221.

Malkesman, O., Asaf, T., Shbiro, L., Goldstein, A., Maayan, R., Weizman, A., Kinor, N., Okun, E., Sredni, B., Yadid, G., Weller, A. (2009). Monoamines, BDNF, Dehydroepiandrosterone, DHEA(S)ulfate, and Childhood Depression-An Animal Model Study. Advances in Pharmacological Sciences 2009.

Maninger, N., Wolkowitz, O. M., Reus, V.I., Epel, E. S., Mellon, S. H. (2009). Neurobiological and Neuropsychiatric Effects of Dehydroepiandrosterone (DHEA) and DHEA Sulfate (DHEAS). Frontiers in Neuroendocrinology 30(1): 65-91.

Markopoulou, K., Papadopoulos, A., [Juruena, M. F](http://www.ncbi.nlm.nih.gov/pubmed/?term=Juruena%20MF%5BAuthor%5D&cauthor=true&cauthor_uid=18805642)., [Poon, L](http://www.ncbi.nlm.nih.gov/pubmed/?term=Poon%20L%5BAuthor%5D&cauthor=true&cauthor_uid=18805642)., [Pariante, C. M](http://www.ncbi.nlm.nih.gov/pubmed/?term=Pariante%20CM%5BAuthor%5D&cauthor=true&cauthor_uid=18805642)., [Cleare, A. J](http://www.ncbi.nlm.nih.gov/pubmed/?term=Cleare%20AJ%5BAuthor%5D&cauthor=true&cauthor_uid=18805642). (2009). The ratio of cortisol/DHEA in treatment resistant depression. Psychoneuroendocrinology 34(1): 19-26.

Maurice, T., Roman, F. J., Privat, A. (1996). Modulation by neurosteroids of the in vivo (+)-[3H]SKF-10,047 binding to sigma 1 receptor in the mouse forebrain. Journal of Neuroscience Research 46(6): 734-743.

Mazat, L., Lafont, S., Berr, C., Debuire, B., Tessier, J. F., Dartigues, J. F., Baulieu, E. E. (2001). Prospective measurements of dehydroepiandrosterone sulfate in a cohort of elderly subjects: Relationship to gender, subjective health, smoking habits, and 10-year mortality. Proceedings of the National Academy of Sciences of the United States of America 98(14): 8145-8150.

McEwen, B. S., Biron, C. A., Brunson, K. W., Bulloch, K., Chambers, W. H., Dhabhar, F. S., Goldfarb, R. H., Kitson, R. P., Miller, A. H., Spencer, R. L., Weiss, J. M. (1997). The role of adrenocorticoids as modulators of immune function in health and disease: neural, endocrine and immune interactions. . Brain Research Reviews 23: 79-133.

Michikawa, T., Nishiwaki, Y., Nakano, M., Iwasawa, S., Yamada, M., Asakura, K., Yoshioka, N., and E. Kuwahara, Takebayashi, T. (2013). Higher Serum Dehydroepiandrosterone Sulfate Levels Are Protectively Associated with Depressive Symptoms in Men, But Not in Women: A Community-Based Cohort Study of Older Japanese. The American Journal of Geriatric Psychiatry 21(11): 1154-1163.

Mocking, R. J., Pellikaan, C. M., Lok, A., Assies, J., Ruhé, H. G., Koeter, M. W., Visser, I., Bockting, C. L., Olff, M., Schene, A. H. (2015). DHEAS and cortisol/DHEAS-ratio in recurrent depression: State, or trait predicting 10-year recurrence? Psychoneuroendocrinology 59: 91-101.

Moriguchi, S., Shinoda, Y., Yamamoto, Y., Sasaki, Y., Miyajima, K., Tagashira, H., Fukunaga, K. (2013). Stimulation of the sigma-1 receptor by DHEA enchances synaptic efficacy and neurogenesis in the hippocampal dentate gyrus of olfactory bulbectomized mice. PLoS One 8(4).

Morita, T., Senzaki, K., Ishihara, R., Umeda, K., Iwata, N., Nagai, T., Hida, H., Nabeshima, T., Yukawa, K., Ozaki, N., Noda, Y. (2014). Plasma dehydroepiandrosterone sulfate levels in patients with major depressive disorder correlate with remission during treatment with antidepressants. Human Psychopharmacology 29(3): 280-286.

Morrison, M. F., Redei, E., TenHave, T., Parmelee, P., Boyce, A. A., Sinha, P. S., Katz, I. R. (2000). Dehydroepiandrosterone sulfate and psychiatric measures in a frail, elderly residential care population. Biological Psychiatry 47(2): 144-150.

Morrison, M. F., Ten Have, T., Freeman, E. W., Sammel, M. D., Grisso, J. A. (2001). DHEA-S levels and depressive symptoms in a cohort of African American and Caucasian women in the late reproductive years. Biological Psychiatry 50(9): 705-711.

Morsink, L. F., Vogelzangs, N., Nicklas, B. J., Beekman, A. T., Satterfield, S., Rubin, S. M., Yaffe, K., Simonsick, E., Newman, A. B., Kritchevsky, S. B., Penninx, B. W. (2007). Associations between sex steroid hormone levels and depressive symptoms in elderly men and women: results from the Health ABC study. Psychoneuroendocrinology 32(8-10): 874-883.

ó Hartaigh, B., Loerbroks, A., Thomas, G. N., Engeland, C. G., Hollands, M. A., Fischer, J. E., Bosch, J. A. (2012). Age-dependent and -independent associations between depression, anxiety, DHEAS, and cortisol: from the MIPH Industrial Cohort Studies (MICS). Psychoneuroendocrinology 37(7): 929-936.

Orentreich, N., Brind, J. L., Vogelman, J. H., Andres, R., Baldwin, H. (1992). Long-term longitudinal measurements of plasma dehydroepiandrosterone sulfate in normal men. Journal of Clinical Endocrinology and Metabolism 75(4): 1002-1004.

Nagata, C., Shimizu, H., Takami, R., Hayashi, M., Takeda, N., Yasuda, K. (2000). Serum concentrations of estradiol and dehydroepiandrosterone sulfate and soy product intake in relation to psychologic well-being in peri- and postmenopausal Japanese women. Metabolism 49(12): 1561-1564.

Phillips, A. C., Batty, G. D., Gale, C. R., Lord, J. M., Arlt, W., Carroll, D. (2011). Major depressive disorder, generalised anxiety disorder, and their comorbidity: associations with cortisol in the Vietnam Experience Study. Psychoneuroendocrinology 36(5): 682-690.

Radloff, L. S. (1977). The CES-D Scale A Self-Report Depression Scale for Research in the General Population Applied Psychological Measurement 1(3): 385-401.

Robichaud, M., Debonnel, G. (2004). Modulation of the firing activity of female dorsal raphe nucleus serotonergic neurons by neuroactive steroids. The Journal Of Endocrinology 182(1): 11-21.

Samaras, N., Samaras, D., Frangos, E., Forster, A., Philippe, J. (2013). A review of age-related dehydroepiandrosterone decline and its association with well-known geriatric syndromes: is treatment beneficial? Rejuvenation Research 16(4): 285-294.

Sierksma, A., Sarkola, T., Eriksson, C. J., van der Gaag, M. S., Grobbee, D. E., Hendriks, H. F. (2004). Effect of moderate alcohol consumption on plasma dehydroepiandrosterone sulfate, testosterone, and estradiol levels in middle-aged men and postmenopausal women: a diet-controlled intervention study. Alcoholism, Clinical and Experimental Research 28(5): 780-785.

Steel N, Huppert FA, McWilliams B, Melzer D. (2003). Physical and cognitive function. In: Marmot M, Banks J, Blundell R, Lessof C, Nazroo J , editors. Health, Wealth and Lifestyles of the Older Population in England: The 2002 English Longitudinal Study Of Ageing. London: Institute for Fiscal Studies.

Steffick, D. E. (2000). HRS/AHEAD Documentation Report. Documentation of affective functioning measures in the Health and Retirement Study. DR-005.

Steptoe, A., Breeze, E., Banks, J., Nazroo, J. (2013). Cohort Profile: The English Longitudinal Study of Ageing. International Journal of Epidemiology 42(6): 1640-1648.

Sugaya, N., Izawa, S., Saito, K., Shirotsuki, K., Nomura, S., Shimada, H. (2015). Effect of prolonged stress on the adrenal hormones of individuals with irritable bowel syndrome. Biopsychosocial Medicine 9(1). doi: 10.1186/s13030-015-0031-7.

Straub, R. H., Tankó, L. B., Christiansen, C., Larsen, P. J., Jessop, D. S. (2008). Higher physical activity is associated with increased androgens, low interleukin 6 and less aortic calcification in peripheral obese elderly women. Journal of Endocrinology 199(1): 61-68.

Tchernof, A., Labrie, F. (2004). Dehydroepiandrosterone, obesity and cardiovascular disease risk: a review of human studies. European Journal of Endocrinology 151(1): 1-14.

Tilvis, R. S., Kähönen. M., Härkönen, M. (1999). Dehydroepiandrosterone sulfate, diseases and mortality in a general aged population. Aging (Milan, Italy) 11(1): 30-34.

Traish, A. M., Kang, H. P., Saad, F., Guay, A. T. (2011). Dehydroepiandrosterone (DHEA)--a precursor steroid or an active hormone in human physiology. The Journal of Sexual Medicine 8(11): 2960-2982.

T’Sjoen, G. G., De Vos, S., Goemaere, S., Van Pottelbergh, I., Dierick, M., Van Heeringen, C., Kaufman, J. M. (2005). Sex steroid level, androgen receptor polymorphism, and depressive symptoms in healthy elderly men. Journal of the American Geriatrics Society 53(4): 636-642.

Turvey, C. L., Wallace, R. B., Herzog, R. (1999). A Revised CES-D Measure of Depressive Symptoms and a DSM-Based Measure of Major Depressive Episodes in the Elderly. International Psychogeriatrics 11(2): 139-48.

Veronese, N., De Rui, M., Bolzetta, F., Zambon, S., Corti, M. C., Baggio, G., Toffanello, E. D., Crepaldi, G., Perissinotto, E., Manzato, E., Sergi, G. (2015). Serum Dehydroepiandrosterone Sulfate and Incident Depression in the Elderly: The Pro.V.A. Study. The American Journal of Geriatric Psychiatry 23(8): 863-871.

WHO (2009). Global health risks: Mortality and burden of disease attributable to selected major risks. World Health Organization.

Wolkowitz, O. M., Reus, V. I., Roberts, E., Manfredi, F., Chan, T., Raum, W. J., Ormiston, S., Johnson, R., Canick, J., Brizendine, L., Weingartner, H. (1997). Dehydroepiandrosterone (DHEA) treatment of depression. Biological Psychiatry 41(3): 311-318.

Wong, S. Y., Leung, J. C., Kwok, T., Ohlsson, C., Vandenput, L., Leung, P. C., Woo, J. (2011). Low DHEAS levels are associated with depressive symptoms in elderly Chinese men: results from a large study. Asian Journal of Andrology 13(6): 898-902.

Yaffe, K., Ettinger, B., Pressman, A., Seeley, D., Whooley, M., Schaefer, C., Cummings, S. (1998). Neuropsychiatric function and dehydroepiandrosterone sulfate in elderly women: a prospective study. Biological Psychiatry 43(9): 694-700.

Zarrouf, F. A., Artz, S., Griffith, J., Sirbu, C., Kommor, M. (2009). Testosterone and depression: systematic review and meta-analysis. Journal of Psychiatric Practice 15(4): 289-305.

**Figure Legends**

**Figure 1.** Analytical Sample Flowchart

**Table Legends**

**Table 1.** Demographic characteristics of older men and women at baseline.

**Table 2.** DHEA(S) and depressive symptoms are negatively associated at baseline (n = 3,083).

**Table 3.** Low DHEA(S) levels predict depressive symptoms at 4-year follow-up (n = 3,009).

**Supplementary Table 1**A) DHEA(S) and depressive symptoms association at baseline in men (n = 1,405) and 1B) in women (n = 1,678).

**Supplementary Table 2**A) DHEA(S) and depressive symptoms at 4-year follow-up in men (n = 1,373) 2B) in women (n = 1,636).

**Figure 1.**

**
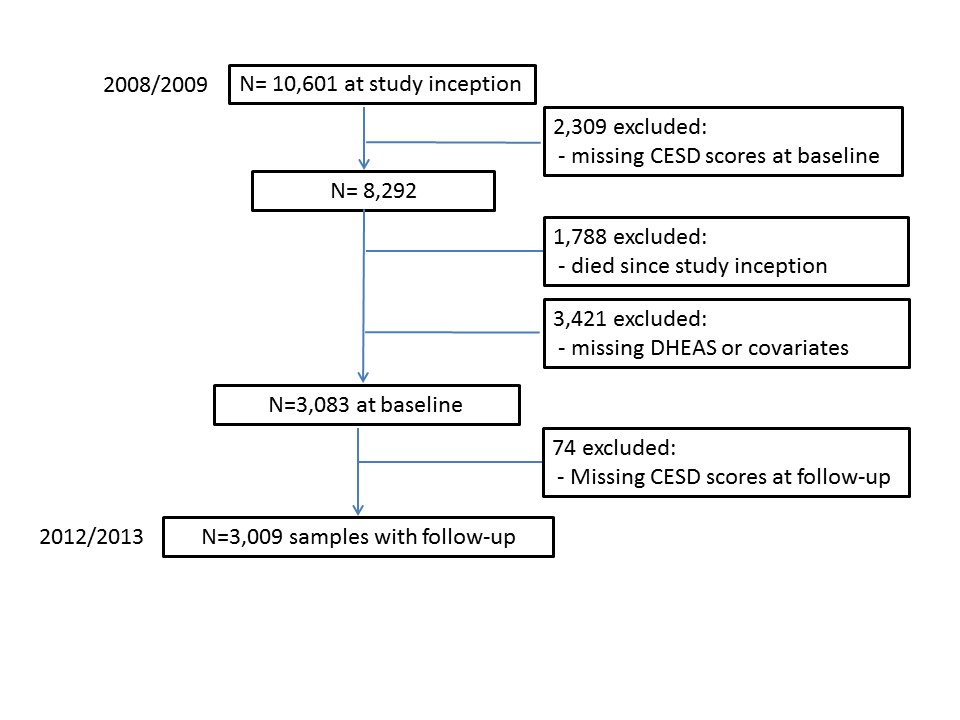
**

**Table 1.**

|  |  |  |  |
| --- | --- | --- | --- |
| **Demographics** |  |  | |
|  |  |  | |
| Age (yrs) (mean ± SD) |  | 65.8 ± 8.7 | |
|  |  |  | |
| Male/Female |  | 46% / 54% | |
|  |  |  | |
| Wealth (%) |  |  | |
|  | Poorest tertile | 34% | |
|  | Tertile 2 | 20% | |
|  | Richest tertile | 45% | |
|  |  |  | |
| BMI (kg/m^2^) (mean ± SD) |  | 28.1 ± 4.9 | |
|  |  |  | |
| Smoking status (%) | current smoker | 11% | |
|  |  |  | |
| Chronic illness (%) |  | 16% | |
|  |  |  | |
| Physical Exercise |  |  | |
|  | Sedentary | 2% | |
|  | Low | 10% | |
|  | Moderate | 52% | |
|  | vigorous | 36% | |
| Alcohol consumption |  |  | |
|  | Never | 35% | |
|  | Occasionally | 48% | |
|  | Daily | 17% | |
|  |  |  | |
| Depression at Baseline |  | 11% | |
|  |  |  | |
| Depression at 4-year follow-up |  | 10% | |
|  |  |  | |
| DHEA(S) (μmol/L) (mean ± SD) |  | 2.41 ± 1.80 | |

_BMI = body mass index;_ _DHEA(S) = dehydroepiandrosterone sulphate._

**Table 2.**

| Current depressive symptoms | B (95% CI) | P-value |
| --- | --- | --- |
| Model 1 | -0.252 (-0.451, -0.052) | 0.014 |
| Model 2 | -0.233 (-0.431, -0.035) | 0.021 |
| Model 3 | -0.200 (-0.397, -0.002) | 0.047 |
| Model 4 | -0.185 (-0.382, 0.013) | 0.067 |
| Model 5 | -0.160 (-0.357-0.036) | 0.109 |

_B = unstandardized beta; CI = confidence interval._ _DHEA(S) = dehydroepiandrosterone-sulphate. Model 1 = DHEA(S), age, sex, antidepressant use, cohabitation status_. _Model 2 = In addition to model 1, adjustment for wealth._ _Model 3 = In addition to model 2, adjustment for cognitive impairment. Model 4 = In addition to model 3, adjustment for chronic illness. Model 5 = In addition to model 4, adjustment for smoking status, physical activity, alcohol consumption and BMI._

**Table 3.**

| Incident depressive symptoms | B (95% CI) | P-value |
| --- | --- | --- |
| Model 1 | -0.338 (-0.525, -0.152) | < 0.001 |
| Model 2 | -0.333 (-0.519, -0.147) | < 0.001 |
| Model 3 | -0.328 (-0.514, -0.142) | 0.001 |
| Model 4 | -0.331 (-0.518, -0.145) | 0.001 |
| Model 5 | -0.332 (-0.520, -0.145) | 0.001 |

_B = unstandardized beta; CI = confidence interval._ _DHEA(S) = dehydroepiandrosterone-sulphate. Model 1 = DHEA(S), age, sex, antidepressant use, cohabitation status_. _Model 2 = In addition to model 1, adjustment for wealth._ _Model 3 = In addition to model 2, adjustment for cognitive impairment. Model 4 = In addition to model 3, adjustment for chronic illness. Model 5 = In addition to model 4, adjustment for smoking status, physical activity, alcohol consumption and BMI._

**Supplementary table 1A**

| Current depressive symptoms –  Men (n = 1405) | B (95% CI) | P-value |
| --- | --- | --- |
| Model 1 | -0.326 (-0.589, -0.064) | 0.015 |
| Model 2 | -0.290 (-0.551, -0.029) | 0.030 |
| Model 3 | -0.271 (-0.532, -0.009) | 0.042 |
| Model 4 | -0.235 (-0.496, 0.027) | 0.079 |
| Model 5 | -0.177 (-0.441, 0.086) | 0.187 |

**Supplementary table 1B**

| Current depressive symptoms –  Women (n = 1678) | B (95% CI) | P-value |
| --- | --- | --- |
| Model 1 | -0.211 (-0.501, 0.079) | 0.153 |
| Model 2 | -0.215 (-0.502, 0.072) | 0.142 |
| Model 3 | -0.201 (-0.488, 0.086) | 0.169 |
| Model 4 | -0.176 (-0.462, 0.109) | 0.226 |
| Model 5 | -0.169 (-0.452, 0.113) | 0.239 |

_B = unstandardized beta; CI = confidence interval._ _DHEA(S) = dehydroepiandrosterone-sulphate. Model 1 = DHEA(S), age, sex, antidepressant use, cohabitation status_. _Model 2 = In addition to model 1, adjustment for wealth._ _Model 3 = In addition to model 2, adjustment for cognitive impairment. Model 4 = In addition to model 3, adjustment for chronic illness. Model 5 = In addition to model 4, adjustment for smoking status, physical activity, alcohol consumption and BMI._

**Supplementary table 2A**

| Incident depressive symptoms –  Men (n = 1405) | B (95% CI) | P-value |
| --- | --- | --- |
| Model 1 | -0.427 (-0.687, -0.166) | 0.001 |
| Model 2 | -0.404 (-0.664, -0.144) | 0.002 |
| Model 3 | -0.392 (-0.653, -0.132) | 0.003 |
| Model 4 | -0.393 (-0.653, -0.132) | 0.003 |
| Model 5 | -0.391 (-0.656, -0.125) | 0.004 |

**Supplementary table 2B**

| Incident depressive symptoms –  Women (n = 1678) | B (95% CI) | P-value |
| --- | --- | --- |
| Model 1 | -0.252 (-0.547, -0.021) | 0.034 |
| Model 2 | -0.233 (-0.550, -0.025) | 0.032 |
| Model 3 | -0.200 (-0.550, -0.024) | 0.033 |
| Model 4 | -0.185 (-0.554, -0.027) | 0.031 |
| Model 5 | -0.160 (-0.567, -0.039) | 0.025 |

_B = unstandardized beta; CI = confidence interval._ _DHEA(S) = dehydroepiandrosterone-sulphate. Model 1 = DHEA(S), age, sex, antidepressant use, cohabitation status_. _Model 2 = In addition to model 1, adjustment for wealth._ _Model 3 = In addition to model 2, adjustment for cognitive impairment. Model 4 = In addition to model 3, adjustment for chronic illness. Model 5 = In addition to model 4, adjustment for smoking status, physical activity, alcohol consumption and BMI._
